# A Semi-Empirical Model to Estimate Maximum Floc Size in a Turbulent Flow

**DOI:** 10.3390/molecules27175550

**Published:** 2022-08-29

**Authors:** Mohamed Bizi

**Affiliations:** BRGM, Water, Environment, Processes Development & Analysis Division 3, BRGM, Avenue C. Guillemin, CEDEX 2, 45060 Orleans, France; m.bizi@brgm.fr; Tel.: +33(0)2-38-64-36-62

**Keywords:** aggregation, flocculation, fractal, Kolmogorov scale, erosion, breakage, mechanistic model, velocity gradient, turbidity

## Abstract

The basic model for agglomerate breakage under the effect of hydrodynamic stress (d_max_ = C.G^−^^γ^) is only applicable for low velocity gradients (<500 s^−1^) and is often used for shear rates that are not representative of the global phenomenon. This paper presents a semi-empirical model that is able to predict mean floc size in a very broad shear range spanning from aggregation to floc fragmentation. Theoretical details and modifications relating to the orthokinetic flocculation output are also provided. Modelling changes in turbidity in relation to the velocity gradient with this model offer a mechanistic approach and provide kinetic agglomeration and breakage index k_a_ and k_b_. The floc breakage mode is described by the relationship between the floc size and the Kolmogorov microscale. Shear-related floc restructuring is analysed by monitoring the fractal dimension. These models, as well as those used to determine floc porosity, density and volume fraction, are validated by the experimental results obtained from several flocculation operations conducted on ultrafine kaolin in a 4-litre reactor tank compliant with laws of geometric similarity. The velocity gradient range explored was from 60 to 6000 s^−1^.

## 1. Introduction

Flocculation is a conventional physico-chemical technique used in many industries, particularly in the industrial ore and mineral processing industry, the food, paper and pharmaceutical industries, and, more prevalently, in water treatment (drinking water and wastewater) [1,2,3,4]. It is a process through which solid or microbial elementary particles dispersed in a fluid agglomerate in the form of “flocs” under the influence of a chemical reactant. The collisions between these particles may be generated by thermal agitation, turbulent agitation or an external force field. In industrial contexts, flocculation is induced by turbulent hydrodynamic conditions. Its efficiency is measured mainly by the degree of clarification of the treated water and by the floc settling rate. The level of these efficiency criteria depends on the physico-chemical characteristics of the conditioning environment, hydrodynamic factors which lead to a high aggregation rate, increased floc size and the formation of optimally textured flocs, and the strength of the floc’s bonds in relation to the hydrodynamic forces encountered. The flocculation process is generally monitored by measuring turbidity and particle size distribution. These parameters are, of course, dependent on the hydrodynamic conditions and the physico-chemical parameters of the surrounding environment. Floc size is directly related to floc porosity, density and fractal dimension. The basic models for turbulence-induced flocculation taken from the work of Argaman et al. [5] and Parker et al. [6] used thus far for the purpose of optimising flocculation are:

(1) $$\frac{dn}{dt}=-k_{a}nG+k_{b}n_{0}G^{p}$$

(2) $$d_{max}=CG^{-\gamma }\;$$

where *n*_0_ is the initial number concentration of primary particles per unit volume; *n* is the number concentration of primary particles per unit volume at time t; *G* is the velocity gradient; *k_a_* is is an index of particle aggregation rate; *k_b_* is an index of floc breakage and erosion rate; *d_max_* is the maximum floc diameter; *C* is the floc strength coefficient that depends on both properties of the floc and fluid and the exponent γ depends on both the breakup mode and size regime of eddies that cause disruption [7]. γ and *p* are similar. (*k_a_nG*) is the aggregation function; (*k_b_n*_0_*G^P^*) is the break-up and erosion function. *k_a_* and *k_b_* depend on the physico-chemical properties of the suspension and the nature of the flocculant used [1,8].

The first model (Equation (1)) is often used to determine the orthokinetic flocculation output *n*_0_/*n_nf_*, assuming that the concentration of the number of residual non-flocculated primary particles (*n_nf_*) is proportional to the turbidity of the supernatant, without taking into account the proportionality constants that can be used to calculate the turbidity from the number of particles. The effect of this approximation may be detrimental to the determination of the kinetic agglomeration and breakage index *k_a_* and *k_b_*. The second model (Equation (2)) for agglomerate breakage under the effect of hydrodynamic stress is only applicable for low velocity gradients (<500 s^−1^) and in the shear ranges produced by jar tests. In order to conduct comprehensive monitoring of the different flocculation phases in terms of turbidity and size in a sufficiently broad velocity gradient range, more suitable models must be developed. This research is entirely in line with this objective.

This paper focuses on the physical aspects of flocculation. The first section consists of a theory chapter that (i) introduces textural models designed to describe floc porosity, density and volume fraction, (ii) describes new developments of semi-empirical relationships suited to the modelling of turbidity and the maximum mean floc diameter in a very broad velocity gradient range, and (iii) presents a flocculation reactor tank that complies with the laws of geometrical similarity that can be transposed to different scales. The second section concerns the application and interpretation of the results, with a review of the main parameters of floc texture and modelling using the proposed semi-empirical relationships. The flocculation operations were carried out on ultrafine kaolin powder in a 4-litre reactor tank with a constant flocculant dosage and a constant conditioning time. The velocity gradients explored ranged from 60 to 6000 s^−1^.

## 2. Theory

Flocculation is a dynamic process whose efficiency is dependent on the physical and physico-chemical properties of the product involved, the physico-chemical characteristics of the environment and the hydrodynamic conditions surrounding floc formation. The main macroscopic structural characteristics that can be used to distinguish flocs include volume, size, porosity, permeability, density, fractal dimension and structure. These different properties are of course interdependent. The fractal dimension represents the mean compactness of ramified or wispy objects. It is based on a statistical arrangement of primary particles to form an aggregate (a floc). This statistical property is independent of the scale of observation. In this study, this dimension is determined either through correlations between certain parameters specific to the flocs, or based on the intensity of the light scattered by the flocs through laser diffraction analysis [9]. The number of primary particles in a floc with mass m and diameter d is provided by the following equation [9,10,11,12,13]:

(3) $$\frac{m_{F}}{m_{P}}=n_{p}=k_{\alpha }{\left(\frac{d_{F}}{d_{P}}\right)}^{D_{f}}$$

where *m_F_* and *d_F_* are the floc mass and diameter, *m_P_* and *d_P_* are the mass and diameter of the primary particle which contributes to the formation of the floc, *D_f_* is the mass fractal dimension of the floc and *k_α_* is the prefactor. The latter depends on parameters including the floc compactness, in other words on its fractal dimension and the size and concentration of primary particles. This prefactor is equal to the base prefactor *k*_0_ when the diameter of gyration is considered to be the diameter of the floc, otherwise, *k_α_* = *k*_0_.α^*D_f_*^. Equation (3) provides the expressions of floc porosity, density and relative volume fraction [4,9]:

(4) $$\varepsilon_{F}=1-k_{\alpha }{\left(\frac{d_{F}}{d_{P}}\right)}^{D_{f}-3}$$

(5) $$\rho_{F}=\rho_{L}+\left(\rho_{S}-\rho_{L}\right)k_{\alpha }{\left(\frac{d_{F}}{d_{P}}\right)}^{D_{f}-3}$$

In the presence of a monodisperse suspension of flocs in which all the primary particles are flocculated (or if not, the number of non-flocculated particles is negligible compared to the number of flocs), the volume fraction relationship can be written as follows:

(6) $$\Phi_{FP}=\frac{1}{k_{\alpha }}{\left(\frac{d_{F}}{d_{P}}\right)}^{3-D_{F}}$$

where ε*_F_* is the average floc porosity; *ρ* is the average density (F: floc, L: liquid, S: solid); Φ*_F_* is the volume fraction of the flocs; Φ*_P_* is the volume fraction of the initial primary particles; Φ*_FP_* is the relative volume fraction of flocs (Φ*_FP_* = Φ*_F_*/Φ*_P_*).

The diameters of the primary particles and flocs considered in this paper are the mean diameters of spheres of equivalent volume.

Based on the analysis of light scattering by the flocs in suspension, it is possible to determine the size distribution, the diameter of gyration and fractal dimension [2,4,9,14]. For the fractal aggregates (flocs) and in the range of validity of the Rayleigh-Gans-Debye approximation, it has been shown that the total scattered intensity I(q) presents power– law dependence in relation to the amplitude of the scattering wave vector *q* [15,16,17]:

(7) For    πRg≪q≪πrP,     I(q) ∝ q−Df

where *R_g_* is the radius of gyration of floc, *r_P_* is the primary particle radius and *D_f_* is the mass fractal dimension of floc. *q* is the magnitude of the scattering wave vector. *q* is a function of the incident beam wavelength (λ = 632.8 nm), the refractive index of the scattering medium and the scattering angle. The textural information is obtained from the analysis of the modulus variation of the scattering wave vector in the *q*-range of 1.81 × 10^−3^ to 4.53 × 10^−1^ µm^−1^ of the Mastersizer S granulometer long bench version (1000 mm lens − 32 detectors).

The fractal dimension is determined in the range *π*/*R_g_* << *q* << *π*/*r_P_* from the negative slope of the linear region of the logarithmic plot of *I(q)* versus *q*. Experimental observation showed that this dimension might have values ranging between 1.4 and 2.8 [18,19]. The radius of gyration can be determined in the Guinier region of the scattering curve from the slope of the linear part of the logarithm plotted for scattered intensity I as a function of *q*^2^ where *qR_g_* << 1 (very low *q* values) using the Guinier approximation [14]:

(8) $$I=I_{0}exp\left(-\frac{R_{g}^{2}}{3}q^{2}\right)$$

**Figure 1:**
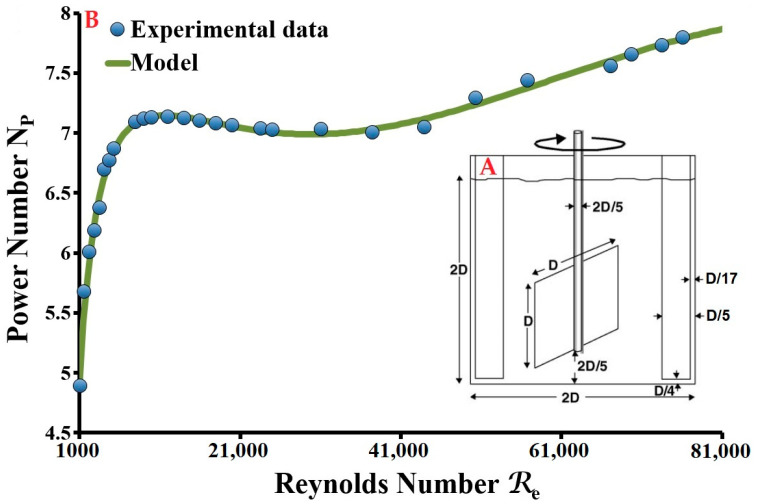
Laboratory conditioning system. (**A**) Geometrical configuration of the reactor; (**B**) Power diagram of the single-blade sheet-type agitator.

Under certain physico-chemical conditions, floc formation, structuring and transformation are directly dependent on the hydrodynamic conditions and in particular on the applied velocity gradients and the nature of the eddies that form during orthokinetic flocculation. These transformations were examined through experiments conducted using a cylindrical flocculation reactor tank equipped with four orthogonal baffles and a single-blade impeller, in the form of a solid square sheet (Figure 1A). The configuration of this reactor tank validates the laws of geometric similarity; only the diameter of impeller D is imposed (D = 87.5 mm, V = 4.2 L, liquid depth = tank diameter). The power *P* and speed of rotation *N* of the impeller, to obtain value G, are determined from the power curve for the flocculation reactor tank used (Figure 1B). This power curve describes the variation in power number *N_P_* as a function of the Reynolds number *R_e_*. It is produced experimentally by measuring the torque applied to the impeller’s drive shaft at different velocities, and possibly different viscosities and densities of the stirred fluid. By modelling this curve, we were able to establish the following empirical function: 

For 1000 ≤ *R_e_* ≤ 100000:

(9) $$N_{P}=P_{1}+P_{2}R_{e}+P_{3}R_{e}^{P_{4}}+P_{5}R_{e}lnR_{e}+P_{6}\frac{R_{e}}{lnR_{e}}$$

*P*_1_ = 0.93002471, *P*_2_ = −0.04623452, *P*_3_ = −2.738211 × 10^−6^, *P*_4_ = 1.5310496,

*P*_5_ = 0.0023565875 and *P*_6_ = 0.23500056

In addition [20]:

(10) $$N_{P}=\frac{P}{\rho_{p}N^{3}D^{5}}$$

(11) $$P=VG^{2}\eta$$

(12) $$R_{e}=\frac{\rho_{p}ND^{2}}{\eta }$$

*V* is the agitated volume; *G* is the velocity gradient; *η* is the dynamic viscosity of the flocculated suspension; *N_P_* is the power number; *P* is the power dissipated by the agitator into the fluid; *D* is the impeller diameter; *N* is the rotational speed of the agitator; *ρ_p_* is the density of the suspension; *R_e_* is the Reynolds number.

Subsequently, the velocity gradient *G*, Reynolds number *R_e_* and power *P* dissipated by the impeller are determined for a given rotation speed by solving the combination of this set of equations using Mathcad. The value for the dynamic viscosity of the flocculated suspension is considered to be independent of the velocity gradient; it is estimated using the Thomas equation [21]:

(13) $$\eta =\eta_{0}\left(1+2.5\emptyset_{F}+10.05\emptyset_{F}{}^{2}+0.062exp\left(\frac{1.875\emptyset_{F}}{1-1.595\emptyset_{F}}\right)\right)$$

where ∅*_F_* is the volume concentration of flocs in the pulp, and η_0_ is the dynamic viscosity of water at 20 °C. The ratio between the volume of the flocs and that of the suspension represents the volume concentration ∅*_F_* occupied by the flocs in the flocculated suspension with the initial solids concentration *C_S_*. This volume concentration is determined by the expression [2]:

(14) ∅F=CSρS1−εF

where *ρ_S_* is the solid density and *ε_F_* is the average floc porosity.

Our previous work in this area [2,4,9] provides more information on the determination of volume concentration, porosity and density of flocs.

The mixing time of the flocculant in the stirred volume is estimated using the empirical Mersmann equation [22]:

(15) $$t_{m}={\left(300\rho_{p}\frac{D_{i}^{5}}{P}\right)}^{1/3}$$

where *ρ_p_* is the density of the suspension; *D_i_* is the internal diameter of the flocculation reactor; *P* is the power dissipated by the agitator into the fluid.

In turbulent environments, flocs are shaped by eddy movements and breakdowns. The interactions between large eddies and between these eddies and the liquid generate smaller, more energetic eddies which, when destroyed, release their energy by viscous dissipation. According to Kolmogorov’s theory of local isotropy, for large Reynolds numbers, the smallest eddies are independent of the overall fluid movement and are isotropic and in statistical equilibrium [23,24]. A recent study into the analysis of local turbulence in a conventional jar test (stirred-tank reactor) [25] reaffirmed that the main dissipation of kinetic energy is due to turbulence-induced viscous dissipation associated with Kolmogorov microscale eddies (small eddies). The mean dimension of this microscale of turbulence (α) is determined based on the mean viscous dissipation rate of turbulent kinetic energy (*E*) and the kinematic viscosity of the mixture (*ν*) [6,26,27]:

(16) $$\alpha ={\left(\frac{\nu^{3}}{E}\right)}^{1/4}$$

According to Kolmogorov’s theory, the rate of energy dissipation of turbulent kinetic energy *E* is deduced from the velocity gradient under mean shear stress *G* [28]:

(17) $$E=\nu G^{2}$$

The impact of shear stress on the main modes of floc disaggregation (splitting and/or erosion) is generally characterised by floc size in relation to the dimension of the turbulence microscale.

The flocculation efficiency of a number of primary particles is evaluated principally as a function of water quality (residual turbidity), the state of the sediment obtained following treatment, and also by the rapidity of the physico-chemical process applied. For a given material and water, these three evaluation criteria depend on the type of reactant used, the hydrodynamic conditions, and consequently, the texture of the flocs formed. The decrease over time in the number of primary particles in the suspension is evaluated by a population balance resulting from a combination of floc aggregation, erosion and splitting functions. On the basis of the work of Argaman et al. [5] and Parker et al. [6], this decrease is expressed by Equation (1). This general equation is a first-order linear differential equation with constant coefficients of the form y′ = ay + b. Its solution is based on the solution of the homogeneous equation y′ = ay and the initial condition is given at *t* = 0, where *n* = *n*_0_. By doing so, we obtain:

(18) $$\frac{n_{nf}}{n_{0}}=\frac{k_{b}}{k_{a}}G^{p-1}+\left(1-\frac{k_{b}}{k_{a}}G^{p-1}\right)exp\left(-k_{a}Gt\right)$$

where *n*_0_ is the number of initial primary particles before flocculation and *n_nf_* is the number of unflocculated primary particles.

Based on this relationship, we propose the following transformations in order to determine *k_a_*, *k_b_* and *p*. Following flocculation, by measuring the residual turbidity we are able to determine the number of non-flocculated particles. Assuming that the residual suspension is monodispersed with *n_nf_* particles per unit volume, i.e., all particles have the same diameter, light attenuation at wavelength λ_0_ on the turbidity meter by *n_nf_* particles is determined by [9,29]:

(19) $$\tau \left(\lambda_{T}\right)=\frac{1}{L}ln\left(\frac{I_{0}}{I_{T}}\right)=n_{nf}C_{ext}$$

The combination of Equations (1) and (18) results in:

(20) $$\tau =n_{0}C_{ext}\left[\frac{k_{b}}{k_{a}}G^{p-1}+\left(1-\frac{k_{b}}{k_{a}}G^{p-1}\right)exp\left(-k_{a}Gt\right)\right]$$

(21) $$=C_{1}\left[C_{2}G^{p-1}+\left(1-C_{2}G^{p-1}\right)exp\left(-C_{3}Gt\right)\right]$$

where τ is the residual turbidity; *I*_0_ is the intensity of the incident light; *I_T_* is the intensity of the transmitted light; *L* is the optical path length; *C_ext_* is the effective extinction cross-section of particles. *C*_1_, *C*_2_, *C*_3_ and *p* are three constants that can be accessed using data processing software (Origin, TableCurve, etc.).

The critical velocity gradient that marks the start of structural reorganisation, reflocculation, or floc erosion during the shear-induced floc fragmentation and erosion phase can be determined using the Quemada model [30]. At equilibrium, in a flocculation range characterised by floc erosion that increases with the velocity gradient G, the variation in the volume fraction Φ*_F_* occupied by the flocs in the suspension, normalised by the volume fraction Φ*_P_* initially occupied by the non-flocculated primary particles, can be modelled using the Quemada relationship [21,30]:

(22) $$\Phi_{FP}=\frac{\Phi_{FP,0}+\Phi_{FP,\infty }{\left(\frac{G}{G_{C}}\right)}^{s}}{1+\Phi_{FP,\infty }{\left(\frac{G}{G_{C}}\right)}^{s}}$$

where *G* is the velocity gradient; *Gc* is the critical velocity gradient average; Φ*_FP_* is the relative volume fraction of flocs (Φ*_FP_* = Φ*_F_*/Φ*_P_*); Φ_*FP*,__0_ is the value of Φ*_FP_* at *G* = 0 s^−1^ (no shear); Φ_*FP*,_*_∞_* is the value of Φ*_FP_* when *G* tends to ∞ (ultimate relative volume fraction); *s* is a parameter that accounts for the shear strength of the flocs.

At a constant concentration of solids, this model only applies above an optimal threshold of *G* (*G*_a/b_) which separates the dominant aggregation range from the erosion and/or splitting range. The value of this threshold depends on the concentration of solids.

Modelling of the global evolution of the mean floc diameter or diameter of gyration as a function of the velocity gradient needs to be specifically developed in this study. Using the dominant process of each phase in the flocculation process and based on Equations (1), (3), (6) and (22), the relationship between the mean diameter and the velocity gradient can be expressed as follows. During the aggregation of primary particles (the first flocculation range), particle decay is expressed as:

(23) $$\frac{dn}{dt}=-k_{a}nG$$

The integration of this relationship provides:

(24) $$n=n_{0}exp\left(-k_{a}Gt\right)$$

In turbulent regimes, the index of particle aggregation rate *k_a_* characterises the collision efficiency. These two parameters are related by the following relationship [1,5,31]:

(25) $$k_{a}=\beta_{T}\sqrt{\frac{24}{5\pi }}\Phi_{F}$$

where Φ*_F_* is the volume fraction of the flocs. *β_T_* is the collision efficiency reflecting the hydrodynamic interaction.

**Figure 2:**
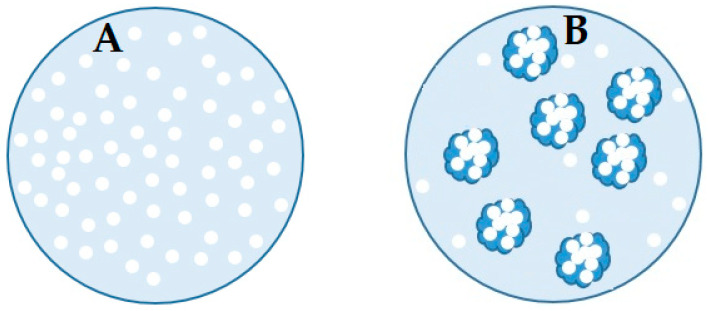
Illustration of the decrease of the number of free primary particles before (**A**) and during flocculation (**B**).

Assuming that the suspensions are composed of particles or flocs with a monodisperse particle size distribution in which the flocs grow at the same rate (Figure 2), the number of primary particles at time 0 is equal at time t to:

(26) $$n_{0}=n_{nf}+n_{j}n_{p}$$

where *n*_0_ is the initial number concentration of primary particles per unit volume; *n_nf_* is the number of unflocculated primary particles; *n_p_* is the number of primary particles in the floc; *n_j_* is the number of flocs (all have the same diameter).

By combining Equations (3), (24) and (26), the equation for the first flocculation phase becomes:

(27) $$\frac{d_{F}}{d_{p}}={\left(\frac{n_{0}}{n_{j}k_{\alpha }}\left[1-exp\left(-k_{a}Gt\right)\right]\right)}^{1/D_{f}^{a}}$$

The evolution of the mean diameter as a function of the velocity gradient during the second phase, during which floc breakage is dominant, can be deduced by combining Equations (6) and (22). The transformation is expressed as:

(28) $$\frac{d_{F}}{d_{p}}={\left(\frac{1}{k_{\alpha }}\frac{\Phi_{FP,0}+\Phi_{FP,\infty }{\left(\frac{G}{G_{C}}\right)}^{s}}{1+\Phi_{FP,\infty }{\left(\frac{G}{G_{C}}\right)}^{s}}\right)}^{1/\left(3-D_{f}^{b}\right)}$$

The previous equation characterises the period dominated by dimensional floc reduction, accounting for the predominance of splitting and erosion over aggregation.

Flocculation is a nonlinear dynamic process divided into two main phases in which formation and/or breakage mechanisms can coexist to different extents. The effects of these mechanisms (Equations (27) and (28)) can therefore be multiplied and translated into the following expression:

(29) $$\frac{d_{F}}{d_{p}}=a{\left(1-exp\left(-k_{a}Gt\right)\right)}^{\beta_{1}}{\left(\frac{b+c{\left(\frac{G}{G_{c}}\right)}^{s}}{1+c{\left(\frac{G}{G_{c}}\right)}^{s}}\right)}^{\beta_{2}}$$

where *d_F_* is the average volume diameter of the flocs; *d_p_* is the diameter of the primary particle; *k_a_* is is an index of particle aggregation rate; *a* is a constant that depends on the initial number of primary particles, the number of flocs, the prefactors *k_α_* and the fractal dimensions; *b* is a constant that depends on the critical dimension separating the two ranges, the initial diameter (*d_0_*), the prefactor *k_α_* and the fractal dimension of the second range; *c* is a constant that depends on the initial diameter (*d_p_*), the ultimate diameter given when *G*→∞, the prefactor *k_α_* and the fractal dimension of the second range; *s* is an exponent whose value is a function of splitting and/or erosion; *β*_1_ = 1/*D_f_^a^* et; *β*_2_ = 1/(3 − *D_f_^b^*). *D_f_^a^* is the average fractal dimension of the flocs formed during the aggregation phase. *D_f_^b^* is the average fractal dimension of the flocs formed during the breaking and/or erosion phase.

## 3. Results and Discussion

The flocculation operations were carried out on ultrafine kaolin in the previously described reactor tank with an anionic flocculant and tap water. The ultrafine kaolin was obtained by hydrocycloning commercial kaolin 7A from the Ploemeur deposit in Morbihan, France. It is composed of around 96% kaolinite and 4% mineral impurities (Illite, Muscovite and Quartz). It had a BET-specific surface area of 17 m²/g, a volume mean diameter of 1.25 µm, an overall negative electric charge, an isoelectric point of around 2.5 and a point of zero charge of 4 [32]. The tap water used had a pH of 8.1 ± 0.1, a conductivity of 352 μS/cm at 25 °C, ionic strength of 5.4 ± 0.4 mmol/L and chemical composition with a dissolved salt concentration of 259 ± 3 mg/L with 3.35 meq/L for cations and 3.51 meq/L for anions. At pH 8, the zeta potential of this ultrafine kaolin was −27 mV in milli-Q water and −10 mV in tap water. The presence of divalent cations Ca^2+^ and Mg^2+^ in tap water decreases the zeta potential. The flocculant used, SNF Floerger AN 934 MPM, is an anionic acrylamide-sodium acrylate copolymer of 35% anionicity and medium molecular weight. This flocculant is commonly used in industrial water treatment processes. Flocculation by this organic polymer occurs due to interparticle bridging following hydrogen bond adsorption of individual polymer chains onto several particles simultaneously, and following neutralisation of the positive sites generated by the adsorption of Ca^2+^ and Mg^2+^ on the kaolinite at pH 8 in tap water, by the carboxyl groups (COO^−^) of the flocculant. These divalent cations form bridges between the anionic surfaces of the kaolin particles and the anionic molecules of the flocculant [33,34].

For each flocculation operation, the flocculant, previously prepared at a concentration of 0.5 g/L, was added using a syringe in a single injection to the pulp which was being stirred. A dosage of 400 g/t (flocculent/kaolin) was used as the optimum value for the three concentrations of solids studied: 6.25 g/L, 12.25 g/L and 25 g/L. Floc growth depends on the stirred volume and the conditioning time, in correlation with the mixing time which increases with the volume, at constant G and constant flocculant dosage. The mixing time estimated by Equation (14) for a velocity gradient between 60 and 5350 s^−1^ increased from 10 to 0.7 s for a volume of 4.2 L for all three concentrations. The imposed flocculant conditioning time was 15 s. The ratio of conditioning time over the estimated theoretical mixing time was between 1.5 and 21. Based on the values of this ratio, it is possible to achieve a homogeneous flocculant distribution in the pulp and efficiency in terms of floc growth, taking into account the stirred volume.

The models cited in the previous paragraph were applied to a series of flocculation operations at different velocity gradients in order to evaluate their ability to macroscopically describe the flocculation efficiency and mechanisms of ultrafine clay particles. Furthermore, the dependence of the model’s parameters on concentration was discussed. The accuracy of each model proposed was assessed using the coefficient of determination *R*^2^ and the normalised relative deviation between the experimental and predicted values Δ*Y*:

(30) $$\Delta Y\left(\%\right)=100\sqrt{\frac{1}{n-p}\sum {\left(\frac{Y_{exp}-Y_{cal}}{Y_{exp}}\right)}^{2}}$$

where *n* is the number of experimental data; *p* is the number of free model parameters; *Y_exp_* and *Y_cal_* are the experimental and model calculated values, respectively.

### 3.1. Turbidity and Flocculation Mechanisms

**Figure 3:**
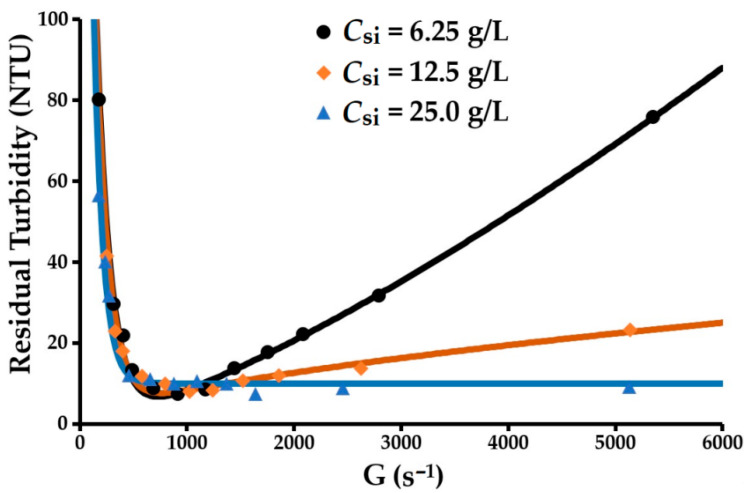
Modeling the variation of residual turbidity vs. velocity gradient.

The evolution of the residual turbidity of the supernatant for the three concentrations of solids as a function of the velocity gradient is presented in Figure 3. The parameters derived from the turbulent flocculation model based on residual turbidity monitoring at a constant temperature (Equation (20)) are presented in Table 1. The model is naturally very faithful to the evolution of the turbidity (*R*^2^ = 0.99, Δ*τ* = 14.2%). The deviations between the experimental and predicted values are acceptable in as far as the turbidity meter used is not very accurate in measuring deviations between very low turbidities. As indicated, the flocculation parameters are affected by the initial concentration of solids *C_Si_* and the shear stress. The linearity between *C_Si_* and minimum residual turbidity indicates that there is a number of particles that escape flocculation and that this number increases with the concentration.

(31) $$\tau_{min}=0.176C_{Si}+5.549,\;R^{2}=0.997$$

The anionic flocculant used immediately induces the formation of large flocs at a relatively low dose of reactant (400 g/t dry kaolin). However, the supernatant shows significant residual turbidity (7 to 9 NTU), even when the flocculant dose is increased. This suggests that the surface chemistry of the mineral impurities in the kaolinite is not suited to that of the flocculant.

The linear increase between the initial concentration of solids *C_si_* and the velocity gradient corresponding to the minimum residual turbidity implies that floc cohesion and the interactions between the primary particles inside the flocs increase with the concentration of solids:

(32) $$G_{\tau_{min}}=50.49C_{Si}+435.97,\;R^{2}=0.99$$

**Figure 4:**
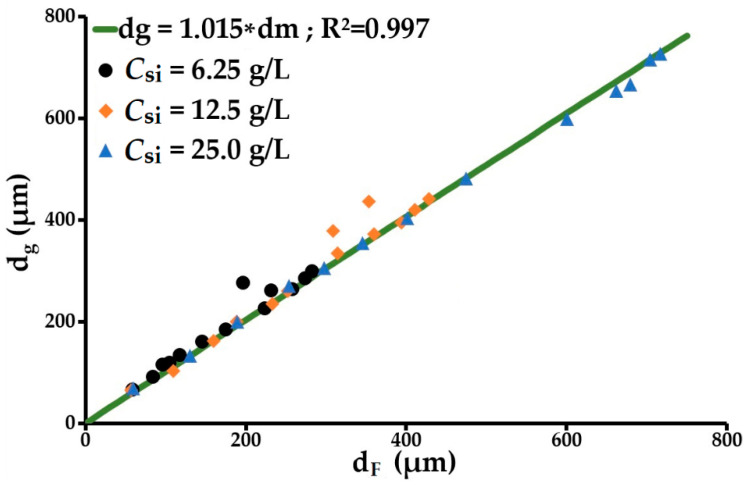
Relationship between the average diameter of flocs and their average gyration diameter.

At 25 g/L and above 900 s^−1^, the residual turbidity changed very little (near-stationary) while the floc size continued to evolve remarkably, showing a decreasing trend according to the velocity gradient applied (later discussed in Section 3.2.6). This phenomenon indicates that floc splitting is dominant at this concentration. The decrease in p as the initial concentration of solids *C_si_* increased can be attributed to the flocs generated at a concentration of 25 g/L being larger than those obtained at 6.25 and 12.5 g/L. The larger floc size at a high *C_si_* value could promote floc fragmentation by splitting over floc erosion. These behaviours imply that p increases as the splitting/erosion ratio increases. A *p* value between 1 and 2.3 may indicate the existence of two parallel floc breakage modes at play. When *p* = 1, splitting is dominant; however, erosion increases as *p* rises. The floc splitting and erosion rate index *k_b_* increases rapidly as *C_si_* rises and *p* decreases. At this level, *k_b_* mainly characterises floc splitting. Conversely, the particle aggregation rate index *k_a_* changes very little with *C_si_*. This also shows that *k_b_* is strongly dependent on the hydrodynamic characteristics of the reactor tank. Floc erosion is higher at 6.25 than at 25 g/L. The dominance of one breakage mode over the other depends on the relative relationship that can exist between floc size and eddy size. In the case of flocs smaller than the turbulence microscale (α), erosion will be the main breakage mode. Conversely, when the floc size is between the microscale and the macroscale, fragmentation by splitting will be the main breakage mode. Before exploring this aspect, we note that this analysis will be performed in relation to the volume mean diameter *d_F_* and the volume diameter d10 for which the cumulative volume function is 10%. These diameters are indicated without any additional processing by the particle size analyser. The volume mean diameter will therefore be favoured over the diameter of gyration. The relationship between these two diameters is shown in Figure 4. The diameters of gyration are determined from the light scattering curve obtained from the Mastersizer S particle size analyzer using the Guinier approximation (Equation (8)). They are 1.4% larger than the mean diameters.

*k_a_* and *k_b_* are the kinetic agglomeration and breakage index; *p* is the floc breakup index that depends on both the breakup mode and size regime of eddies that cause disruption; *τ_min_* is the minimum residual turbidity; *G_τ__min_* (s^−1^) is the velocity gradient corresponding to the minimum residual turbidity.

**Figure 5:**
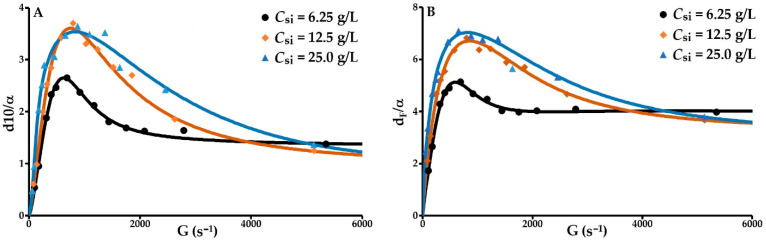
Relative ratio of floc quartile diameter d10. (**A**) and mean floc diameter *d_F_*; (**B**) to Kolmogorov microscale as a function of the velocity gradient.

Floc formation is the outcome of the collision between and cohesion of particles and primary micro-flocs. Floc size is dependent on the relationship between cohesive and hydrodynamic forces. Cohesive forces are related to the nature of the particles (mineralogy, surface chemistry, size, shape and texture), the intrinsic properties of the reactants and the physico-chemical characteristics of the medium. The hydrodynamic forces depend on the geometrical structure of the reactor tank, the stirrer and the mechanical conditions imposed. Under the operating conditions described above, the two variables are the concentration of solids and the velocity gradient. The hydrodynamic forces are therefore analysed via the velocity gradient and its influence on the ratio between floc size and the Kolmogorov microscale (α). This ratio is mostly greater than 1 in the case of d10 and greater than 2 in the case of the mean diameter; therefore, the flocs are mostly in an inertial range where they are exposed to turbulence-induced strains and shear stress (Figure 5). Although the Kolmogorov microscale is far from the primary particle size, it was possible to model the experimental data for these two relationships with Equation (29). The coefficient of determination *R*^2^ = 0.99 for the two relationships, and the normalised relative deviations in relation to the predicted values are 4.8, 5.5 and 5.5% for d10/α as a function of G and 2.4, 2.7 and 4.6% for *d_F_*/α as a function of G.

The flocculation process is divided into three phases. The first phase is dominated by the agglomeration phenomenon. The size of the flocs formed from the primary particles increase rapidly to a maximum size that is subject to a balance between cohesive and hydrodynamic (aggregation/breakage) forces. Phase 2, characterised by a decrease in *d_F_*/α with slopes that are more or less identical for the three concentrations of solids, involves the formation of micro-flocs by fragmentation of the large flocs. The third phase, whose start point depends on the initial concentration of solids, consists of a phase of strong hydrodynamic stress exerted on the surface of the flocs and micro-flocs, resulting in erosion that is inversely proportional to *C_si_*.

### 3.2. Floc Structure

**Figure 6:**
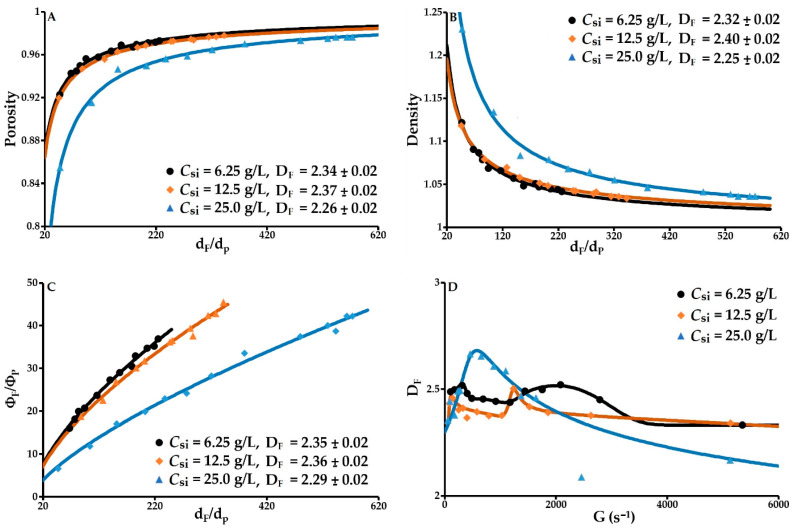
Textural parameters of flocs determined through laser diffractometry. (**A**) Porosity vs. *d_F_/d_P_*; (**B**) Density vs. *d_F_/d_P_*; (**C**) Relative volume fraction of flocs vs. *d_F_/d_P_*; (**D**): Fractal dimension vs. velocity gradient.

In industrial contexts, flocculation efficiency is measured mainly by the degree of clarification of the treated water (elimination of turbidity) and by the floc settling rate. In a turbulent environment, with given physico-chemical characteristics, these two parameters depend on the hydrodynamic factors, which lead to a high aggregation rate, increased floc size and the formation of optimally textured flocs. The texture defined by the floc size, porosity, density, volume fraction and fractal dimension is, therefore, a fundamental parameter to be controlled in order to achieve the objective defined. These interrelated physical characteristics are determined from the particle size analyses [2,9]. Figure 6 show the behaviour of these physical parameters in a range of velocity gradients between 60 and 6000 s^−1^ with a constant flocculant dosage (400 g/t) and constant agitation time (15 s). Each velocity gradient corresponds to mean porosity, density, volume fraction, size and fractal dimension values.

#### 3.2.1. Floc Porosity

Porosity is modelled as a function of *d_F_*/*d_P_* by Equation (4). The coefficient of determination *R*^2^ = 0.99 for the three concentrations of solids (6.25, 12.5 and 25 g/L), and the normalised relative deviations in relation to the predicted values are 0.2, 0.1 and 0.3%. A perfect prediction is obtained with this model. As indicated, porosity increases with floc size due to a primary particle arrangement that traps water (Figure 6A). The higher the floc porosity, the higher their water content will be. This specificity means that flocs will occupy an increasing volume fraction as their size increases (Figure 6C). This phenomenon leads to a gradual reduction in the mean distance between flocs, thus increasing the hydrodynamic surface stress and promoting contact between flocs, which in turn leads to hydrodynamic erosion that increases with the velocity gradient. From 6 to 13 g/L, the porosity is between 0.920 and 0.987. At a concentration of 25 g/L, this parameter drops significantly and lies between 0.855 and 0.980 for 25 g/L. At a constant shear rate, the porosity decreases from a critical volume fraction of primary particles. Flocculation at 25 g/L generates flocs that are significantly more compact than at 6.25 and 12.5 g/L. While the average fractal dimensions obtained using Equation (4) are statistically comparable for the three initial concentrations of solids, the slightly smaller fractal dimension at *C_si_* = 25 g/L is consistent with the decrease in this dimension in the case of density variation as a function of *d_F_*/*d_P_* at *C_si_* = 25 g/L (Figure 6A,B). This can be explained by the formation of primary flocs, which form the final flocs. The prefactors *k_α_* for the three concentrations are 1.0, 0.9 and 2.5 for porosity and 1.0, 0.7 and 2.6 for density respectively. The difference between the values of the *k_α_* parameter of the fractal relationship could reflect the more compact arrangement of the initial particles in the primary flocs at 25 g/L compared to 6.25 and 12.5 g/L.

#### 3.2.2. Floc Density

The mean floc density is modelled by Equation (5). The coefficient of determination *R*^2^ = 0.99 for the three concentrations of solids, and the normalised relative deviations in relation to the predicted values are 0.3, 0.2 and 0.4%; the model is perfectly suited. At all concentrations of solids, this parameter decreases as the mean floc size increases (Figure 6B). This continuous, monotonic decrease with size reflects an increase in the mean porosity. As with porosity, this decrease depends on the volume fraction of the primary particles. At 25 g/L, flocculation produces denser flocs with a significantly lower mean fractal dimension. Low density combined with high porosity is likely to affect the floc settling rate and reduce the flocs’ resistance to hydrodynamic stress.

#### 3.2.3. Volume Fraction vs. Average Diameter

The relative volume fraction of the flocs as a function of *d_F_*/*d_P_* is obtained using Equation (6). The coefficient of determination *R*^2^ = 0.99 for the three concentrations of solids (6.25, 12.5 and 25 g/L), and the normalised relative deviations in relation to the predicted values are 2.3, 2.6 and 4.4%. An excellent prediction is obtained with this model (Figure 6C). The mean fractal dimensions obtained by this model are statistically comparable and follow the same trends observed for porosity and density. Floc growth results in an increase in the flocs’ porosity, a decrease in their density and an increase in their volume concentration. At a constant velocity gradient, the drop in the volume fraction as the concentration of solids rises is due to the increase in the number of primary particles in each floc, resulting in a decrease in porosity and an increase in density.

#### 3.2.4. Fractal Dimension

The evolution of the fractal dimension *D_F_* as a function of the velocity gradient is presented in Figure 6D. With each flocculation operation, the fractal dimension is deduced from the floc diffusion diagram (Equation (7)). This dimension lies between 2.3 and 2.5 for concentrations of 6.25 and 12.5 g/L, and between 2.2 and 2.7 for a concentration of 25 g/L. The latter concentration generates flocs that are significantly more compact. Floc compactness increases with *D_F_*. Above a value of 2.4, flocs can be considered to be dense and compact [4,9,35]. Variation of *D_F_* implies that the flocs formed are sensitive to the shear stress imposed. As expressed in Figure 6D, the fractal dimension contains information on structural changes in the flocs in relation to the velocity gradient and the initial concentration of solids. Changes in state are indicated by the presence of one or more peaks. For the first peak, its rising slope characterises a phase dominated by the aggregation of primary particles and micro-flocs. At its tip, the system is in equilibrium between cohesion and breakage forces. The following drop in the fractal dimension marks the second phase. For concentrations of solids of 6.25 and 12.5 g/L, this phase leads to the formation of micro-flocs through the fragmentation of large flocs, followed by the restructuring of the flocs into a more compact form clearly shown by the presence of a second peak. At the tip of this peak, *D_F_* equals 2.5–2.52. The third phase for these two concentrations of solids is dominated by the splitting of the restructured flocs. At 25 g/L, there is only one peak. The rising slope, as previously mentioned, indicates the dominance of aggregation and the slope after this peak decreases continuously and monotonically with floc breakage being dominant at the beginning and erosion increasing with the velocity gradient. At 25 g/L, erosion becomes dominant from around 2000 s^−1^. The predominant mechanisms depend strongly on the binding forces of the aggregate in relation to the hydrodynamic forces encountered.

#### 3.2.5. Volume Fraction vs. Velocity Gradient

**Figure 7:**
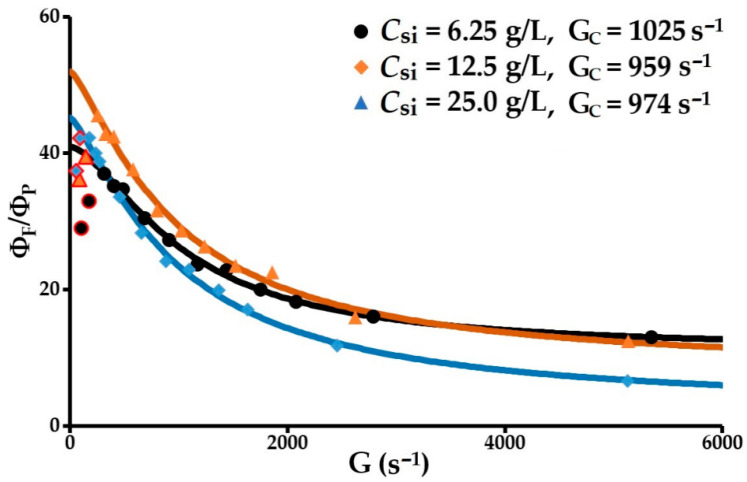
Relative flocs volume fraction vs. the velocity gradient.

The relative volume fraction of the flocs as a function of the velocity gradient is obtained using Equation (22). The coefficient of determination *R*^2^ = 0.99 for the three concentrations of solids (6.25, 12.5 and 25 g/L), and the normalised relative deviations in relation to the predicted values are 2.0, 4.4 and 3.4%. Based on these results, the model is rigorous and is able to determine the critical gradients and ultimate volume fractions (Figure 7). In the given order of the three initial concentrations of solids, the critical gradients are 1025, 959 and 974 s^−1^ respectively. At 6.25 and 12.5 g/L, these gradients correspond to the start point for the restructuring of flocs that have been fragmented and perfectly match the beginning of the second fractal dimension peaks discussed above (Figure 6D). At 25 g/L, the critical gradient at 974 s^−1^ is attributed to the start of erosion. The ultimate diameters, deduced by combining the values of the ultimate relative volume fractions and Equation (4), are, in increasing order of the concentrations of solids, 44.5, 30.3 and 9.3 µm, bearing in mind that the average primary particle size is 1.25 µm. According to these results, the flocculation operation generates very strong micro-flocs.

#### 3.2.6. Modelling of the Evolution of the Mean Floc Diameter vs. Velocity Gradient

**Figure 8:**
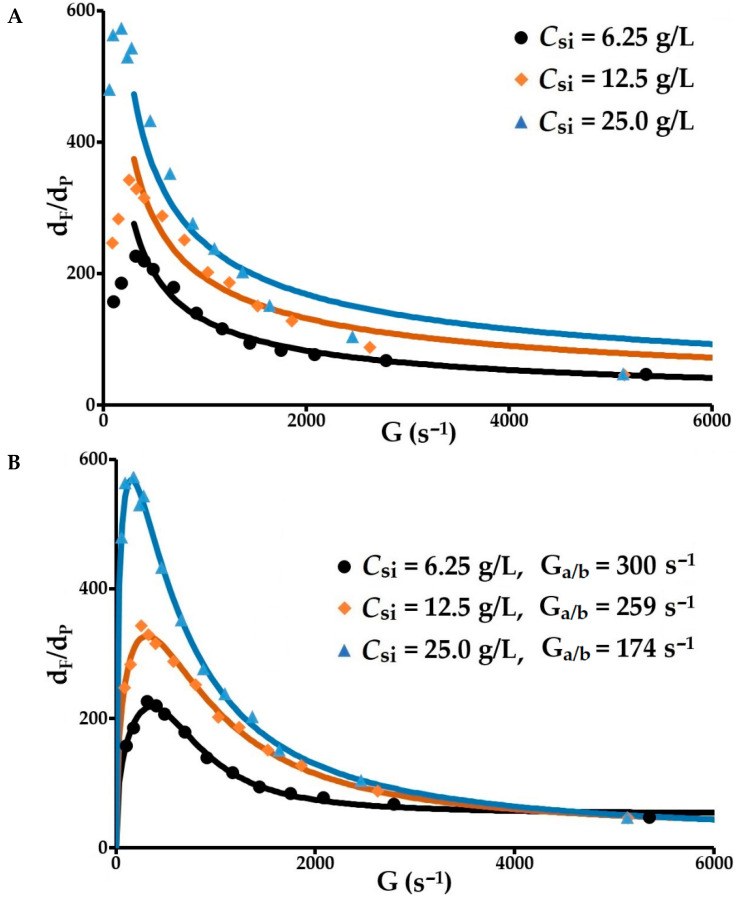
Variation of mean floc diameter with increasing velocity gradient. (**A**) Application of Parker’s model (d_max_ = C.G^−^^γ^); (**B**) Application of the model proposed in this study.

It should be noted that the empirical expression proposed by Parker, d_max_ = C.G^−^^γ^ [6], is not suitable for modelling experimental results in the range for which it was developed (Figure 8A). This model for agglomerate breakage under the effect of hydrodynamic stress is only applicable for low velocity gradients (<500 s^−1^), in particular, in the shear ranges produced by the jar tests. Conversely, the model developed through this research and formulated by Equation (29) applies perfectly and covers the two main flocculation ranges for a very wide range of velocity gradients (Figure 8B). This expression was able to be executed using TableCurve (2D Version 5.01 SYSTAT Software Inc., 2002). With a view to obtaining a global prediction, it seemed appropriate to introduce the mean fractal dimension of the aggregation phase and the fragmentation and erosion phase into the equation developed (Equation (29), β1 and β2). These means are calculated for each concentration of solids from the results obtained from the processing of the light scattering curves measured by the particle size analyser. In this case, the number of parameters in this equation is reduced from eight to six. The main results obtained are presented in Table 2. The coefficient of determination *R*^2^ = 0.99 for the three concentrations of solids (6.25, 12.5 and 25 g/L), and the normalised relative deviations in relation to the predicted values are 7.4, 4.2 and 4.5%. An excellent prediction is obtained with this model.

These results confirm the determining role of the velocity gradient and highlight the following properties. The greater the floc size, the higher the *C_si_* during both the aggregation period and the splitting/erosion period. The variation of this mean size as a function of G reaches a maximum whose abscissa G_a/b_ decreases as *C_si_* increases. The dimensions of the flocs that increase with G when G < G_a/b_ correspond to the dominant aggregation range. Above G_a/b_, floc dimensions decrease as G increases, indicating the dominant presence of splitting and/or erosion in this shear range. The value of this threshold G_a/b_ depends on the initial concentration of solids. Concomitantly, above this threshold, the increase in the velocity gradient leads to a reduction in floc size, volume fraction and porosity, and an increase in density (constant flocculant dosage and constant conditioning time). The decrease in the parameter s as *C_si_* increases is accompanied by an increase in the flocs’ mechanical strength, which accounts for an increase in the density of the bonds between the polymer and particle surface.

In the proposed model (Equation (29)), the particle aggregation rate index *k_a_* is assumed to be constant during the conditioning period. This hypothesis is an approximation because *k_a_* depends on the floc dimensions and the arrangement of the particles within the flocs, in relation to an aggregate density and a collision radius that are dependent on the fractal dimension. This hypothesis steers the research towards the determination of an adjustment value *k_a_* which may be far from the mechanistic value close to the actual value provided in Table 1. Interpretation becomes difficult but the order of magnitude is given. The model is able to very accurately predict the maximum diameter *d_max_*, the velocity gradient for the transition between the dominant phases G_a/b_ and the parameter indicating floc shear strength s.

## 4. Materials and Methods

The flocculation operations were conducted on the ultrafine fraction of commercially available kaolin 7A from the Ploemeur deposit in the Morbihan area of Brittany, France. This kaolin is generally used in paper coating. The particle-size distribution of this ultrafine kaolin was measured by helium-neon laser diffractometry (632.8 nm) using the Malvern Mastersizer S particle size analyser (Malvern Panalytical, Malvern, UK) in its basic configuration (short bench with a 2.4 mm optical path). Its mineralogical composition was determined by X-ray diffraction using the BRUKER D8 Advance Da Vinci diffractometer (Bruker France S.A.S, Champs-sur-Marne, France) and its BET specific surface area was determined at 77 K from the nitrogen adsorption isotherm obtained with the Micromeritics ASAP 2050 batch sorptometer (Micromeritics Instrument Corp., Norcross, GA, USA).

**Figure 9:**
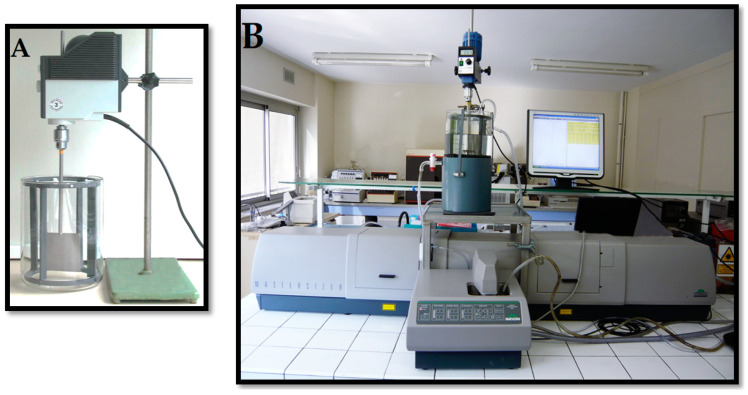
(**A**) photo of the flocculation reactor; (**B**) photo of the setup implemented for floc texture analysis.

Flocculation operations were conducted in a 4.2-litre reactor tank fitted with four orthogonal riffles and a single-blade sheet-type impeller. With this impeller, it is possible to reach high velocity gradients at fairly low rotation speeds while decreasing the turbulence heterogeneity in the stirred volume (Figure 9A). This flocculation tank configuration relatively accurately reproduces the hydrodynamic characteristics of an industrial flocculation box [2]. Several flocculation operations were performed between 60 and 6000 s^−1^. A short 15-s conditioning period with a commercial anionic flocculant AN 934 MPM at a concentration of 400 g/t was applied to each flocculation operation. The size and texture of the flocs (porosity, density, fractal dimension and volume fraction) were determined by laser diffraction with the same Malvern MasterSizer S particle size analyser but using its long bench, a 10 mm optical path measurement unit and a gravity-fed stream via a reactor tank also with a capacity of 4.2-litres, fitted with four orthogonal riffles, placed on top of the particle size analyser (Figure 9B). At the end of each flocculation operation performed at a given velocity gradient, a sample of the flocculated suspension was sampled with a calibrated ladle (known volume and concentration). This floc sample was carefully placed in the reactor tank of the particle size analyser. The new floc suspension, diluted to a measurement concentration for which the attenuation effects of the central laser beam do not exceed 28% obscuration [9], was gently stirred (G ≤ 20 s^−1^) by an impeller identical to the flocculation impeller. With this configuration, it was possible to determine the particle size distributions between 4.2 and 3473 µm. It eliminates recirculation of the suspension by pump during measurements, as per the conventional configuration of the particle size analyser, which significantly affects floc size and texture. After a 1min settling period in the flocculation reactor tank, the residual turbidity of the supernatant was measured using the Turb 555 IR turbidity meter (*λ*_T_ = 860 nm). Each sample was taken at a depth of 3 cm.

## 5. Conclusions

In light of the experimental results, the semi-empirical models introduced or developed in this paper account for the main mechanisms involved in the flocculation of ultrafine mineral particles under excessive shear stress.

For given physico-chemical characteristics, and the three concentrations of solids between 6 and 25 g/L, the residual turbidity, porosity, density, volume fraction and mean floc size were rigorously modelled. The residual turbidity model combining floc aggregation and breakage can readily determine the agglomeration and breakage kinetic index *k_a_* and *k_b_*. With the model for floc aggregation and breakage under the effect of hydrodynamic stress developed through this research, it possible to predict the maximum diameter *d_max_*, the velocity gradient G_a/b_ that separates the aggregation phase from the floc breakage and/or erosion phase, and the parameter indicating floc shear strength “s”. This model shows that the flocs’ mechanical strength increases with increasing concentration. Floc growth results in an increase in the flocs’ porosity, a decrease in their density and an increase in their volume concentration.

The flocs’ fractal dimensions—estimated from the fractal relationships between their density, porosity or volume fraction and their mean size—are homogeneous. The variation in the fractal dimension deduced from the floc diffusion diagrams as a function of the velocity gradient clearly points to the restructuring of flocs produced during the floc fragmentation phase. The fractal dimension can therefore provide important structural information on floc density and compactness and in particular on their restructuring period.

The turbidity, size, density, porosity and fractal dimension can thus be considered as control parameters for the optimisation of the efficiency of the flocculation processes as a whole. Flocculation efficiency can only be optimised by measuring and modelling these characteristics in relation to the shear rates applied.

## Figures and Tables

**Table 1:** Results of applying the proposed model to the analysis of residual turbidity variation with velocity gradient.

*C_si_* (g/L)	*k*_a_	*k_b_* (s)	p	*τ_min_* (NTU)	*G_τmin_* (s^−1^)	*R* ^2^	Δ*τ* (%)
6.25	4.99 × 10^−4^	1.47 × 10^−9^	2.32	6.7	726	0.997	14.0
12.5	5.35 × 10^−4^	1.91 × 10^−7^	1.62	7.6	1105	0.997	14.0
25	7.65 × 10^−4^	1.89 × 10^−5^	1.00	9.3	1621	0.998	14.2

**Table 2:** Parameters from the proposed model (Equation (29)) for the relationship between floc size and velocity gradient.

Csi (g/L)	D_F_^a^	D_F_^b^	G_a/b_ (s^−1^)	k_a_	s
6.25	2.5	2.46	300	0.60 × 10^−4^	2.28
12.5	2.44	2.39	259	0.20 × 10^−4^	1.48
25	2.41	2.47	174	0.25 × 10^−4^	1.13

## Data Availability

Not applicable.
